# The Preparation of Ginsenoside Rg5, Its Antitumor Activity against Breast Cancer Cells and Its Targeting of PI3K

**DOI:** 10.3390/nu12010246

**Published:** 2020-01-18

**Authors:** Yannan Liu, Daidi Fan

**Affiliations:** 1 Shaanxi Key Laboratory of Degradable Biomedical Materials, School of Chemical Engineering, Northwest University, 229 North Taibai Road, Xi’an 710069, Shaanxi, China; liuyannan_2009@163.com; 2 Shaanxi R&D Center of Biomaterials and Fermentation Engineering, School of Chemical Engineering, Northwest University, 229 North Taibai Road, Xi’an 710069, Shaanxi, China; 3 Biotech. & Biomed. Reserch Institute, Northwest University, Taibai North Road 229, Xi’an 710069, Shaanxi, China

**Keywords:** ginsenoside Rg5, MCF-7 cells, PI3K, apoptosis, autophagy

## Abstract

Ginsenosides have been reported to possess various pharmacological effects, including anticancer effects. Nevertheless, there are few reports about the antitumor activity and mechanisms of ginsenoside Rg5 against breast cancer cells. In the present study, the major ginsenoside Rb1 was transformed into the rare ginsenoside Rg5 through enzymatic bioconversion and successive acid-assisted high temperature and pressure processing. Ginsenosides Rb1, Rg3, and Rg5 were investigated for their antitumor effects against five human cancer cell lines via the MTT assay. Among them, Rg5 exhibited the greatest cytotoxicity against breast cancer. Moreover, Rg5 remarkably suppressed breast cancer cell proliferation through mitochondria-mediated apoptosis and autophagic cell death. LC3B-GFP/Lysotracker and mRFP-EGFP-LC3B were utilized to show that Rg5 induced autophagosome-lysosome fusion. Western blot assays further illustrated that Rg5 decreased the phosphorylation levels of PI3K, Akt, mTOR, and Bad and suppressed the PI3K/Akt signaling pathway in breast cancer. Moreover, Rg5-induced apoptosis and autophagy could be dramatically strengthened by the PI3K/Akt inhibitor LY294002. Finally, a molecular docking study demonstrated that Rg5 could bind to the active pocket of PI3K. Collectively, our results revealed that Rg5 could be a potential therapeutic agent for breast cancer treatment.

## 1. Introduction

Breast cancer remains the most frequent malignancy and has become the leading cause of cancer-related deaths among females worldwide [1]. Breast cancer accounted for almost 30% of the total new cancer diagnoses and 14% of cancer mortalities in females, in 2018 [2]. Currently, chemotherapy is one of the major treatments for breast cancer [3]. Nevertheless, poor aqueous solubility, chemotherapeutic resistance, and side effects hinder the therapeutic application of chemotherapeutics [4,5]. Therefore, it is imperative to seek more effective and novel natural drugs against breast cancer to minimize the side effects of conventional chemotherapy.

The use of herbal medicines as alternative antitumor agents showing hypotoxicity has attracted substantial attention [6]. Ginsenosides are the major active chemical constituents of ginseng, and more than 100 ginsenosides have been identified [7]. It has been extensively used as a traditional herbal medicine in Asian countries to promote health due to its various medicinal functions [8]. Major ginsenosides consist of Rb1, Rb2, Rc, Rd, Re, and Rg1, which account for 90% of the total ginsenoside content in ginseng [9]. However, rare ginsenosides are their deglycosylated forms and these possess greater pharmaceutical potential than the major ginsenosides [10]. Among the rare ginsenosides, ginsenoside Rh2 and compound K exhibit strong antitumor activity via the induction of apoptosis [11,12]. Similarly, it has also been demonstrated that ginsenoside Rg3 remarkably suppresses tumor growth via the EGFR/PI3K/Akt signaling pathway in pancreatic cancer cell lines [13,14]. Additionally, our research groups have demonstrated that ginsenoside Rk3 exerts an obvious inhibitory effect on lung cancer by inducing apoptosis. Ginsenoside Rh4 can trigger apoptosis and autophagic cell death in colorectal cancer without side effects on organ function in mice [15,16].

Ginsenoside Rg5, a rare ginsenoside in ginseng [17], is prepared through the deglycosylation of ginsenoside Rb1 and dehydration of the carbon at position 20 of ginsenoside Rg3. Combined with one sugar moiety, the diol-type ginsenoside Rg5 is characterized by the ability to dissolve more readily in water than other polysaccharide ginsenosides, facilitating its utilization in pharmaceutics. (The logP values for Rg5 and Rg3 are 3.5 and 5.9, respectively; the solubilities of Rg5 and Rg3 in water at room temperature are 503.46 and 60.44 µg/mL, respectively). Nevertheless, due to the difficulty associated with its extraction, little attention has been paid to the effects of Rg5, which could be a promising antineoplastic agent (in an oral formulation or via injection). Recent studies have indicated that Rg5 dramatically suppresses the proliferation of cervical cancer cells by activating apoptosis [17]. However, the antitumor mechanism of Rg5 against breast cancer cells and its potential targeted therapy have rarely been investigated.

In this research, we investigated the antitumor effects of Rg5 on human breast cancer by the triggering of apoptosis and autophagy. Furthermore, the underlying molecular mechanism whereby Rg5 induces its apoptotic and autophagic capacities through the inhibition of the PI3K/Akt signaling pathway in breast cancer was also elucidated. Ultimately, molecular docking was performed to identify PI3K as the antitumor target of Rg5 in breast cancer.

## 2. Materials and Methods

### 2.1. Experimental Materials

RPMI 1640 and penicillin/streptomycin were obtained from HyClone (Logan, UT, USA). Fetal bovine serum was supplied by Biological Industries (Israel). Z-VAD-FMK, 3-MA, and LY294002 were obtained from Selleck (Shanghai, China).

Primary antibodies against cleaved caspase-3, caspase-8, caspase-9 and PARP, AKT, phospho-AKT, mTOR, phospho-mTOR, and phospho-Bad were supplied by AbSci (Vancouver, WA, USA). Primary antibodies against cytochrome-c, Bax, Bcl-2, Atg5, Atg12, Beclin-1, LC3B, P62, PI3K, and Bad were purchased from Proteintech (Chicago, IL, USA). β-actin was purchased from Abcam (Cambridge, UK). Phospho-PI3K was obtained from Abbkine (Sacramento, CA, USA). The secondary antibody to IgG was purchased from Abbkine.

### 2.2. Preparation of Ginsenoside Rg5

Ginsenoside Rb1 was supplied by Chengdu Puruifa Technology (Sichuan, China). Ginsenoside Rb1 was converted to ginsenoside Rg3 using β-glucosidase [18]. Ginsenoside Rg3 was dissolved in 0.25 mol/L citric acid and transformed into ginsenoside Rg5 at 121 °C with high pressure processing for 2 h. The purity of ginsenoside Rg5 was detected using HPLC (SSI, Philadelphia, PA, USA).

Thin layer chromatography (TLC) was performed with silica gel plates (60 F254, Merck, Darmstadt, Germany) with the developing solvent CHCl_3_:CH_3_OH:H_2_O (10:5:1, *v*/*v*/*v*, lower phase). Spots on the TLC plates were detected by spraying plates with 10% H_2_SO_4_ followed by heating at 110 °C for 10 min.

### 2.3. Cell Culture

Human lung cancer cells (NCI-H460), hepatocellular carcinoma cells (SMMC-7721), breast cancer cells (MCF-7), colorectal cancer cells (CACO-2), and gastric cancer cells (SGC-7901) were purchased from the American Type Culture Collection (ATCC, USA). The cell lines were cultured in RPMI 1640 medium in a 5% CO_2_ atmosphere incubator at 37 °C and were provided with 10% fetal bovine serum and 1% penicillin/streptomycin.

### 2.4. Cell Viability Assay

Cell viability was measured by MTT assay. MCF-7 cells were seeded at a density of 5 × 10^3^ cells per well in 96-well plates and treated with various concentrations of Rb1, R-Rg3, S-Rg3, and Rg5. At the indicated time, MTT was added to the wells followed by incubation for 4 h. Afterwards, dimethyl sulfoxide was added to dissolve the formazan crystals. The optical density of the solution at 490 nm was measured using a microplate reader (Biotek, Burlington, VT, USA). The probit model was used to compute IC50 values.

### 2.5. Clone Formation Assay

MCF-7 cells (500 cells/well) were grown in 6-well plates and exposed to Rg5 for 24 h. The medium was changed every second day for two weeks until the cells grew into colonies. Finally, the colonies were fixed using methanol, washed with PBS twice, and stained with Giemsa stain.

### 2.6. Acridine Orange/Ethidium Bromide (AO/EB) Staining Assay

AO and EB were used to stain the live cells and dead or apoptotic cells, respectively. MCF-7 cells (5 × 10^5^ cells per well) were grown in 6-well plates and exposed to Rg5 for 24 h. Afterwards, the cells were stained using AO/EB solution (Solarbio, China) followed by detection using a fluorescence microscope (Nikon, Japan).

### 2.7. Flow Cytometric Analysis of Apoptosis

MCF-7 cells were seeded at a density of 5 × 10^3^ cells per well in 6-well plates and then exposed to Rg5 for 24 h. After the cells were harvested and incubated for 15 min in binding buffer including Annexin V-FITC and PI, they were detected by flow cytometry (Becton Dickson, CA).

### 2.8. Measurement of Mitochondrial Membrane Potential (MMP)

The MMP was measured using a JC-10 assay kit (Beyotime, China). The MCF-7 cells were cultured at a density of 5 × 10^3^ cells per well in 6-well plates and exposed to Rg5 for 24 h. Subsequently, the collected cells were incubated with JC-10 at 37 °C for 20 min, followed by analysis with an inverted fluorescence microscope or flow cytometry. Mitochondrial depolarization was indicated by a decrease in the red/green fluorescence intensity ratio.

### 2.9. Western Blot Assay

MCF-7 cells were lysed in RIPA buffer (Beyotime, China) containing 1 mM phenylmethylsulfonyl fluoride (PMSF) for 20 min on ice. Then, the proteins were separated on SDS-PAGE gels and transferred to PVDF membranes. After blocking with TBST including 5% skim milk, the PVDF membranes were incubated using the primary antibodies at 4 °C overnight followed by incubation with an HRP-conjugated secondary antibody. Detailed specifications for the primary and secondary antibodies are presented in Table 1. Signals were observed by the enhanced chemiluminescence (ECL) substrate (Merck Millipore, Boston, MA, USA) with a Gel Image system (Tanon 5200, Shanghai, China).

### 2.10. Quantitative Real-Time Polymerase Chain Reaction (qRT-PCR)

Total RNA was extracted from the MCF-7 cells by TRIzol reagent (Ambion, MA, USA). cDNA was obtained from the total RNA by a reverse-transcription kit. The cDNA was amplified as follows: 95 °C for 10 min, 40 cycles of amplification at 95 °C for 10 s, 60 °C for 10 s, and 72 °C for 15 s. Data were analyzed by the 2^−ΔΔCT^ method. The primers are shown in Table 2.

### 2.11. MitoTracker Green Assay

The morphology of the mitochondria was assessed using MitoTracker Green (Beyotime, Shanghai, China). The MCF-7 cells (5 × 10^5^ cells per well) were grown in six-well plates and exposed to Rg5 for 24 h. Subsequently, the cells were washed twice with PBS and stained with MitoTracker Green at 37 °C for 30 min, followed by detection with fluorescence microscopy.

### 2.12. Transmission Electronic Microscopy (TEM)

MCF-7 cells (5 × 10^3^ cells per well) were cultured in 6-well plates and exposed to Rg5 for 24 h. The treated cells were harvested and fixed using 2.5% glutaraldehyde. The ultrathin sections were subsequently stained using 1% uranyl acetate and lead citrate. Afterwards, the autophagosome morphology was visualized by TEM (JEM-1230, Tokyo, Japan).

### 2.13. Immunofluorescence Microscopy

Breast cancer cells were subjected to immunofluorescence analysis [19]. Fixed and immunofluorescently stained cells were imaged using a fluorescence microscope (Nikon, Tokyo, Japan). For measurement of the intralysosomal function with LysoTracker Red, MCF-7 cells were seeded in 6-well plates and exposed to Rg5. Afterwards, the cells were washed twice with PBS and stained with LysoTracker Red at 37 °C for 30 min, followed by detection with a fluorescence microscope. For the mRFP-EGFP-LC3B assay, MCF-7 cells were cultured in 6-well plates and transfected with mRFP-EGFP-LC3B (Addgene, 21074, Cambridge, MA, USA) with Lipofectamine 2000 (ThermoFisher Scientific, 11668027, Boston, MA, USA) for 24 h, followed by Rg5 treatment. Subsequently, MCF-7 cells were fixed with 4% paraformaldehyde and analyzed with a fluorescence microscope. Cells with GFP-LC3B+ puncta (green), mRFP-LC3B+ (red), or GFP+ mRFP+ (yellow) puncta were examined, and the experiments were performed in triplicate.

### 2.14. Molecular Docking

The crystal structure of PI3K (PDB code: 3APC) was obtained from the Protein Data Bank. The three-dimensional (3D) structure of Rg5 was drawn via ChemDraw, and the energy was minimized with Chem3D. PI3K and Rg5 were docked with AutoDock Vina 4.2. The protein and ligand were prepared with AutoDock Tools. The docking results were detected using the PyMOL Molecular Graphics System.

### 2.15. ADMET Prediction

ADMET (absorption, distribution, metabolism, excretion, and toxicity) is a prerequisite in drug design and discovery studies since the attributes of molecules play a critical role in the preclinical and clinical phases. PreADMET (http://preadmet.bmdrc.kr/) is a web-based application software program that was used to predict the ADMET properties of Rg5.

### 2.16. Statistical Analysis

The experimental results are shown as the means ± standard deviations (SDs) of three independent experiments. The data were analyzed via Student’s *t*-test with SPSS version 19.0 software (SPSS Inc., Chicago, IL, USA). The statistical variation at the level of *p* < 0.05 was considered to be significant.

## 3. Results

### 3.1. Comparison of the Cytotoxicities of Rb1, R-Rg3, S-Rg3, and Rg5 in Various Tumor Cells

**Figure 1:**
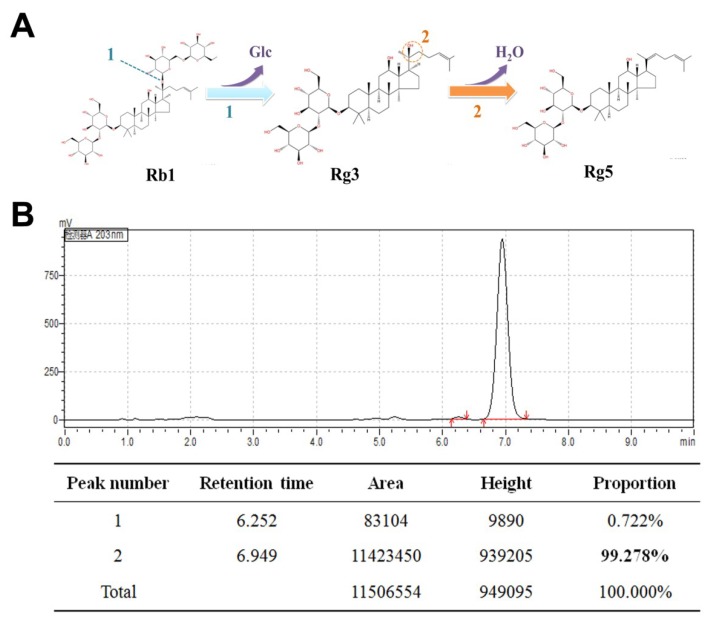
The preparation of ginsenoside Rg5: (**A**) The two steps by which the ginsenoside Rb1 is converted into the ginsenoside Rg5 and (**B**) analytical chromatogram of the obtained ginsenoside Rg5. The bold “99.278%” represents the purity of the separated ginsenoside Rg5.

As shown in Figure 1A, there were two steps for the conversion of ginsenoside Rb1 to Rg5. In the first step, ginsenoside Rb1 was transformed into R-Rg3 and S-Rg3 through an enzymatic bioconversion by deglycosylation at carbon 20. Subsequently, ginsenoside Rg3 was transformed into Rg5 with acid-assisted high temperature and pressure processing by dehydration at carbon 20. TLC analysis showed that ginsenoside Rb1 converted ginsenoside Rg3 within four days using β-glucosidase (Figure S1). Most of the ginsenoside Rg3 was transformed into ginsenoside Rg5 at 121 °C with high-pressure processing within 2 h (Figure S2). Figure 1B reveals that the purity of the separated ginsenoside Rg5 was 99.27%, which was observed through HPLC analysis.

**Figure 2:**
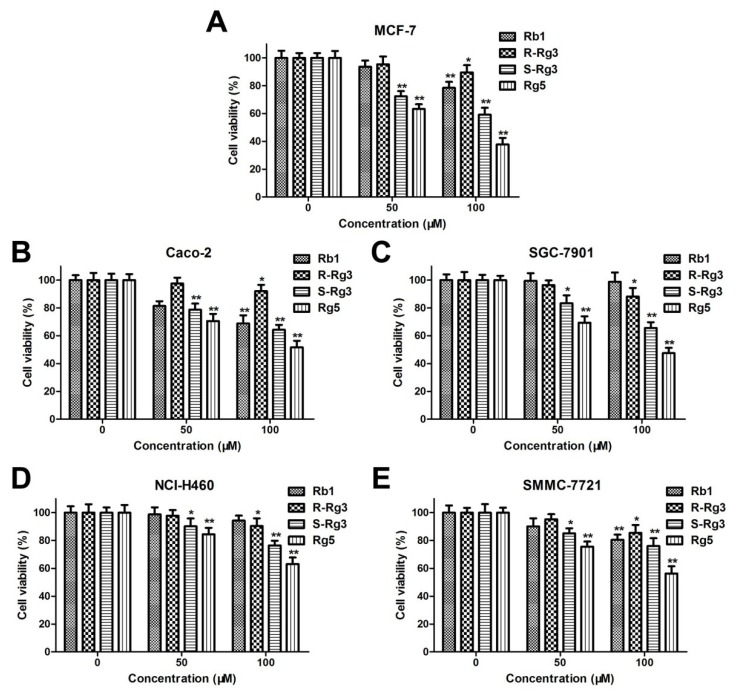
The cytotoxic effects of Rb1, R-Rg3, S-Rg3, and Rg5 on various human cancer cell lines: MCF-7 cells (**A**), CACO-2 cells (**B**), SGC-7901 cells (**C**), NCI-H460 cells (**D**), and SMMC-7721 cells (**E**). * *p* < 0.05 and ** *p* < 0.01 as compared with the control group.

The antiproliferative activities of Rb1, R-Rg3, S-Rg3, and Rg5 on various human cancer cell lines, such as human lung cancer cells (NCI-H460), colorectal cancer cells (CACO-2), hepatocellular carcinoma cells (SMMC-7721), gastric cancer cells (SGC-7901), and breast cancer cells (MCF-7) were evaluated via the MTT assay. As shown in Figure 2A–E, ginsenoside Rb1, R-Rg3, S-Rg3, and Rg5 all decreased the viabilities of different cancer cells in a concentration-dependent manner after 48 h of treatment. Moreover, ginsenoside Rg5 exhibited the greatest cytotoxicity in the various cancer cells among different ginsenosides.

### 3.2. Rg5 Inhibits Breast Cancer Cell Viability

**Figure 3:**
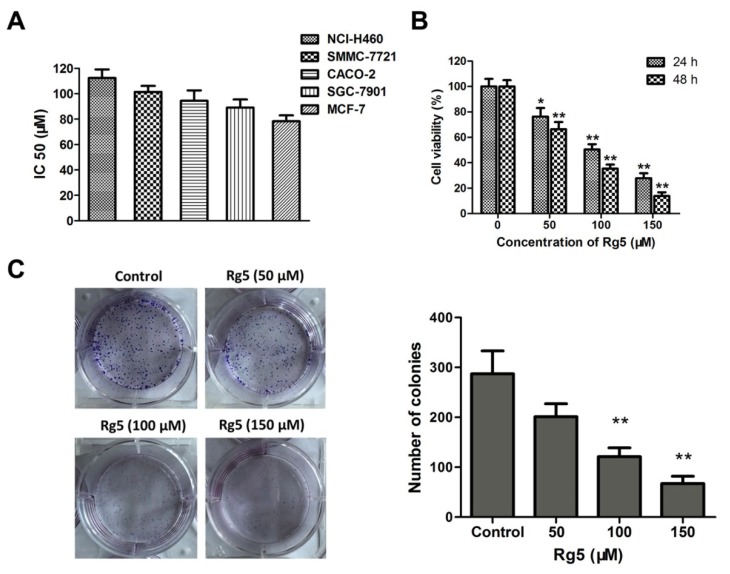
Rg5 suppresses cell viability and colony formation in human breast cancer cells. (**A**) The IC50 values of Rg5 after 48 h treatment were determined in NCI-H460, SMMC-7721, CACO-2, SGC-7901, and MCF-7 cells. (**B**) MCF-7 cells were incubated with Rg5 at different doses (0, 50, 100, and 150 μM) for 24 h and 48 h. Cell viability was detected via MTT assay. (**C**) Colony formation assay of MCF-7 cells with control or Rg5. * *p* < 0.05 and ** *p* < 0.01 as compared with the control group.

The IC50 values in NCI-H460, CACO-2, SMMC-7721, SGC-7901, and MCF-7 cells after 48 h of exposure to Rg5 were 112.32 ± 6.83 μM, 101.46 ± 4.75 μM, 94.52 ± 8.21 μM, 89.09 ± 6.47 μM, and 78.39 ± 4.63 μM, respectively (Figure 3A), and these results demonstrated that Rg5 exhibited the greatest antiproliferative activity against MCF cells among the various cancer cells. Furthermore, MCF-7 cells were exposed to different concentrations of Rg5 for 24 and 48 h. As indicated in Figure 3B, the cell viability of these breast cancer cells significantly decreased in a concentration- and time-dependent fashion after Rg5 exposure. Figure 3C reveals that Rg5 treatment markedly reduced the number of colonies of MCF-7 cells as compared with those in the control. These results strongly suggested that Rg5 inhibited breast cancer cell proliferation in a dose- and time-dependent manner.

### 3.3. Rg5 Induces Caspase-Dependent Apoptosis in Breast Cancer Cells

**Figure 4:**
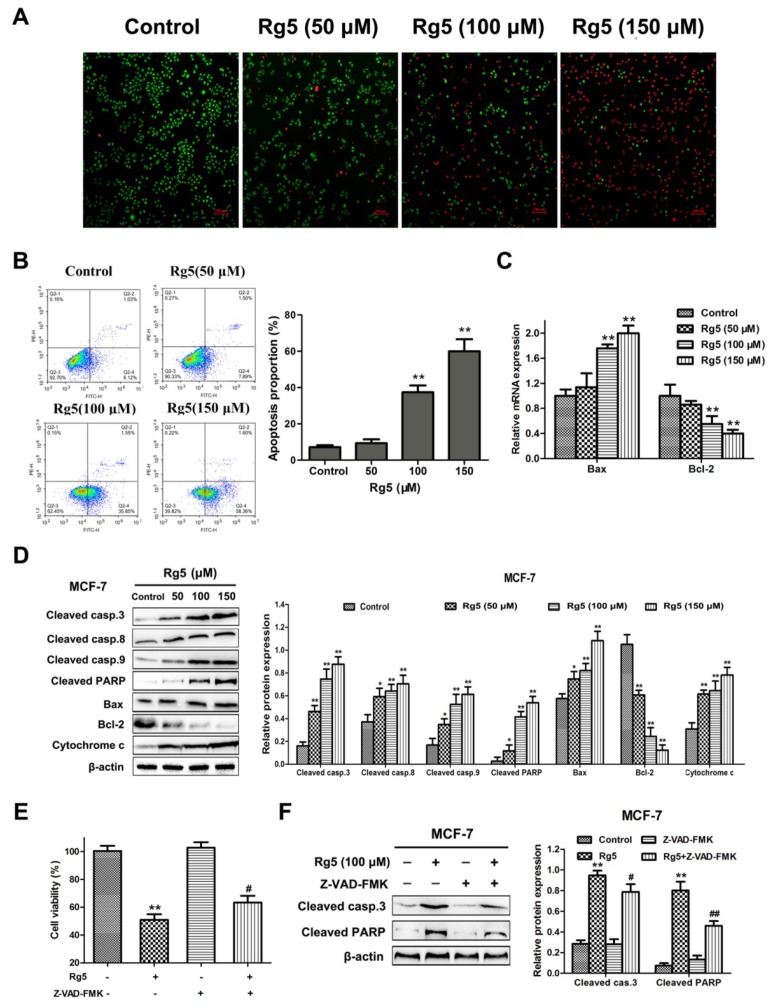
Rg5 triggers apoptosis in human breast cancer cells. (**A**) AO/EB staining was performed to assess apoptosis-induced morphological variation via fluorescence microscopy. Scale bars = 100 μm. (**B−D**) MCF-7 cells were exposed to Rg5 at different concentrations for 24 h. (**B**) Cells were detected by flow cytometry after Annexin V-FITC/PI staining. The chart illustrates the proportion of apoptotic cells. (**C**) The relative mRNA expression levels of Bax and Bcl-2 were determined using real-time qRT-PCR. (**D**) The levels of apoptosis-related proteins were analyzed via Western blot assay. (**E, F**) MCF-7 cells were exposed to Rg5 (100 μM) in the presence of Z-VAD-FMK (100 μM). (**E**) The MTT assay was used to detect cell viability. (**F**) The expression levels of cleaved caspase-3 and cleaved PARP were determined via Western blot analysis. * *p* < 0.05 and ** *p* < 0.01 as compared with the control group. ^#^ *p* < 0.05 and ^##^ *p* < 0.01 as compared with Rg5-treated cells.

To evaluate the effects of Rg5 on apoptosis, AO/EB staining and flow cytometry were investigated in MCF-7 cells. As illustrated in Figure 4A, the number of apoptotic cells was dose-dependently greater in MCF-7 cells induced by Rg5 than in live cells. Flow cytometric analysis with Annexin V-FITC/PI double staining revealed that the proportion of early and late apoptotic cells was remarkably increased in a concentration-dependent way (Figure 4B). Specifically, the rate of apoptotic cells increased from 7.15 ± 1.09% in the control to 59.96 ± 6.64% of the cells treated with 150 μM Rg5.

Subsequently, we explored the expression of important signaling mRNAs and proteins involved in apoptosis via qRT-PCR and Western blotting assays, respectively. Figure 4C indicates that Rg5 clearly increased Bax and decreased Bcl-2 expression at the mRNA level. Consistently, Rg5 treatment led to the upregulation of cleaved caspase-3, cleaved caspase-8. cleaved caspase-9, cleaved PARP, Bax. and cytochrome C and the downregulation of Bcl-2 protein levels in a concentration-dependent fashion (Figure 4D). To further verify the role of caspase activation in Rg5-induced apoptosis, we used the pancaspase inhibitor Z-VAD-FMK to pretreat the cells. As expected, we observed that pretreatment with Z-VAD-FMK attenuated Rg5-induced cell death by the MTT assay (Figure 4E). Furthermore, the Western blot results showed that Z-VAD-FMK remarkably decreased the expression levels of cleaved caspase-3 and cleaved PARP induced by Rg5 (Figure 4F). Collectively, these findings implied that Rg5 triggered caspase-dependent apoptosis in breast cancer cells.

### 3.4. Rg5 Induces Apoptosis via the Mitochondria-Mediated Pathway

**Figure 5:**
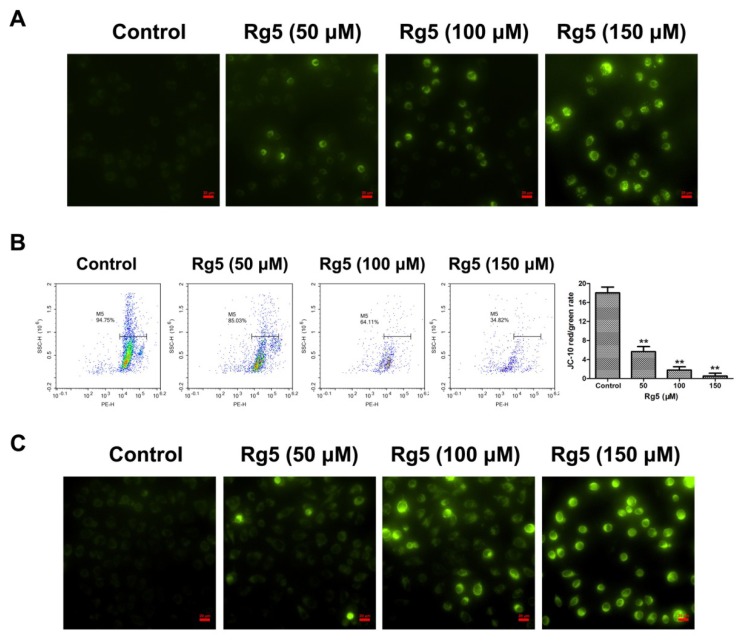
Rg5 induces apoptosis via mitochondria-mediated pathway. MCF-7 cells were exposed to Rg5 at various concentrations for 24 h. The mitochondrial membrane potential was analyzed with JC-10 staining using fluorescence microscopy (**A**) and flow cytometry (**B**). The histogram represents ratio of red/green fluorescence intensity. (**C**) The fluorescence images of MCF-7 cells after mitochondrial staining with MitoTracker Green. Scale bars = 20 μm. ** *p* < 0.01 as compared with the control.

To further explore the apoptotic mechanism induced by Rg5 in breast cancer cells, the MMP was investigated to evaluate mitochondrial integrity. As observed in Figure 5A, a relatively high J-10 monomer green fluorescence was exhibited after exposure to Rg5 as compared with the control group. In agreement with the fluorescence assay, Rg5 quantitatively reduced the JC-10 red/green fluorescent signal in a concentration-dependent manner using flow cytometry (Figure 5B). These results indicated that Rg5 dramatically decreased the MMP in breast cancer cells. Additionally, the mitochondrial morphology in Rg5-treated cells was detected by MitoTracker Green as the mitochondrial stain. Figure 5C shows no green fluorescence in the mitochondria without any treatment; however, enhanced green fluorescence was detected in mitochondria after treatment with various concentrations of Rg5. This result indicated that Rg5 injured the mitochondrial integrity in MCF-7 cells. Taken together, Rg5-induced apoptosis was triggered by the mitochondria-mediated pathway in breast cancer cells.

### 3.5. Rg5 Triggers Autophagy, Which Promotes Cell Death in Breast Cancer Cells

**Figure 6:**
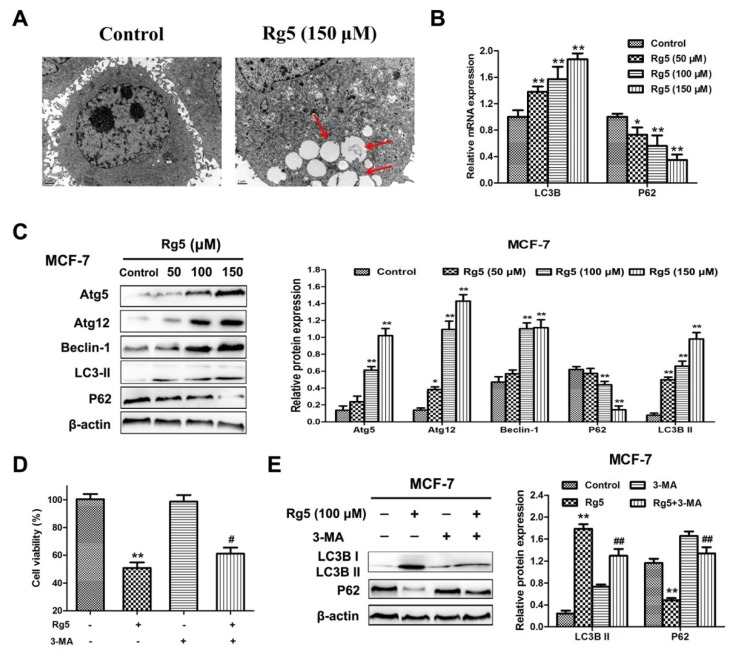
Rg5 triggers autophagy, which leads to cell death. (**A**) Autophagy was detected in MCF-7 cells after Rg5 exposure for 24 h via TEM. Arrows represent autophagosomes. Scale bars = 1 μm. (**B**,**C**) MCF-7 cells were exposed to Rg5 at different doses for 24 h. (**B**) The relative mRNA expression levels of LC3B and p62 were performed via real-time qRT-PCR. (**C**) Western blotting was performed to evaluate the expression of autophagy-related proteins. (**D**,**E**) MCF-7 cells were exposed to Rg5 (100 μM) in the presence of 3-MA (2 mM). (**D**) Cell viability was detected via MTT assay. (**E**) Expression levels of the LC3BII and p62 proteins were performed using Western blot analysis. * *p* < 0.05 and ** *p* < 0.01 as compared with the control group; ^#^ *p* < 0.05 and ^##^ *p* < 0.01 as compared with Rg5-trated cells.

We, then, examined whether Rg5 triggered autophagy in breast cancer cells. First, a TEM assay was used to detect the intracellular morphologic changes in MCF-7 cells. As shown in Figure 6A, autophagosomes formed in Rg5-treated cells as compared with the control. Moreover, we analyzed the expression of autophagy-related mRNAs and proteins using qRT-PCR and Western blotting assays, respectively. Figure 6B reveals that Rg5 significantly promoted LC3B and inhibited p62 expression at the mRNA level. In agreement with the results of the mRNA assay, the protein expression levels of Atg5, Atg12, Beclin-1, and LC3B-II were obviously elevated, and the protein expression level of p62 was remarkably decreased in response to Rg5 treatment (Figure 6C).

To investigate the role of autophagy induced by Rg5 as pro-survival or pro-death, MCF-7 cells were exposed to Rg5 in the presence of the autophagy inhibitor 3-MA. The MTT assay indicated that pretreatment with 3-MA weakened the cell death induced by Rg5 (Figure 6D). Furthermore, Western blot results revealed that 3-MA obviously reduced the level of LC3B-II and increased the level of p62 after Rg5 exposure (Figure 6F). Overall, the above results demonstrated that Rg5 induced autophagy, which exerted a pro-death effect in breast cancer cells.

### 3.6. Rg5 Augments the Fusion of Autophagosomes and Lysosomes in Breast Cancer Cells

**Figure 7:**
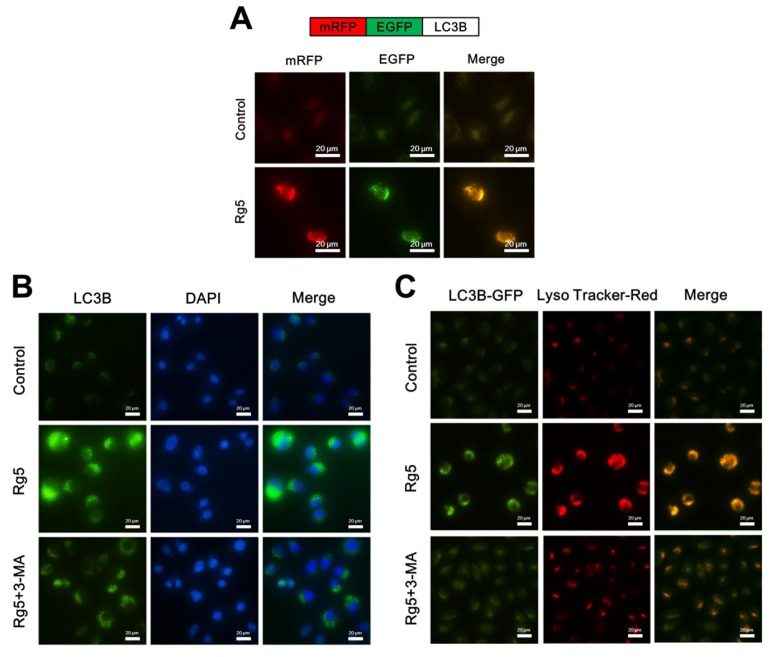
Rg5 augments the fusion of autophagosomes and lysosomes in human breast cancer cells. (**A**) Upper panel represents the schematic diagram of tfLC3B. MCF-7 cells were transfected with tfLC3B-encoding plasmid and treated with 100 μM Rg5. Representative images are shown. (**B**) MCF-7 cells were treated with 100 μM Rg5 in combination with 2 mM 3-MA and subjected to immunofluorescence analyses for LC3B. Representative images are shown. (**C**) MCF-7 cells were transfected with GFP-LC3B-encoding plasmid, treated with 100 μM Rg5 in combination with 2 mM 3-MA, and stained with LysoTracker-Red. Representative fluorescence images are shown. Scale bars = 20 μm.

Redistribution of LC3B from the cytosol to autophagosomes indicated the formation of autophagosomes [20]. To investigate whether Rg5 induces autophagosomes in MCF-7 cells, we transfected MCF-7 cells with the tandem fluorescent-tagged LC3B plasmid mRFP-EGFP-LC3B, which is used to identify autophagosomes (GFP-positive and RFP-positive merge as yellow) and autophagolysosomes (GFP-negative and RFP-positive merge as red) [21]. Figure 7A shows that MCF-7 cells presented an enhancement in both red and yellow fluorescence upon Rg5 exposure, indicating increased autophagosome formation. Furthermore, immunofluorescence analyses of Rg5-treated MCF-7 cells indicated a larger number of LC3B puncta formation, while control cells presented diffuse green fluorescence in the cytosol (Figure 7B). To further examine whether Rg5 induces autophagic flux, MCF-7 cells were treated with Rg5 in combination with the autophagic inhibitor 3-MA. Figure 7B shows that pretreatment with 3-MA attenuated the green fluorescence in the presence of Rg5, which further indicated that Rg5 increased autophagic flux.

The final stage of autophagy is the fusion of autophagosomes with lysosomes to form autophagolysosomes [20], which was investigated via staining GFP-LC3B-transfected MCF-7 cells with LysoTracker Red (an acidic pH marker for lysosomes). As shown in Figure 7C, compared to the control cells, Rg5-treated MCF-7 cells presented significant overlap between LC3B and lysosomal signals, indicating the formation of autophagolysosomes. Moreover, pretreatment with 3-MA weaken the autophagolysosomes accumulation in the presence of Rg5 (Figure 7C). Taken together, the above results demonstrated that Rg5 increases autophagic flux and the fusion of autophagosomes and lysosomes in breast cancer cells.

### 3.7. Rg5 Triggers Apoptosis and Autophagy by Blocking the PI3K Pathway in Breast Cancer Cells

**Figure 8:**
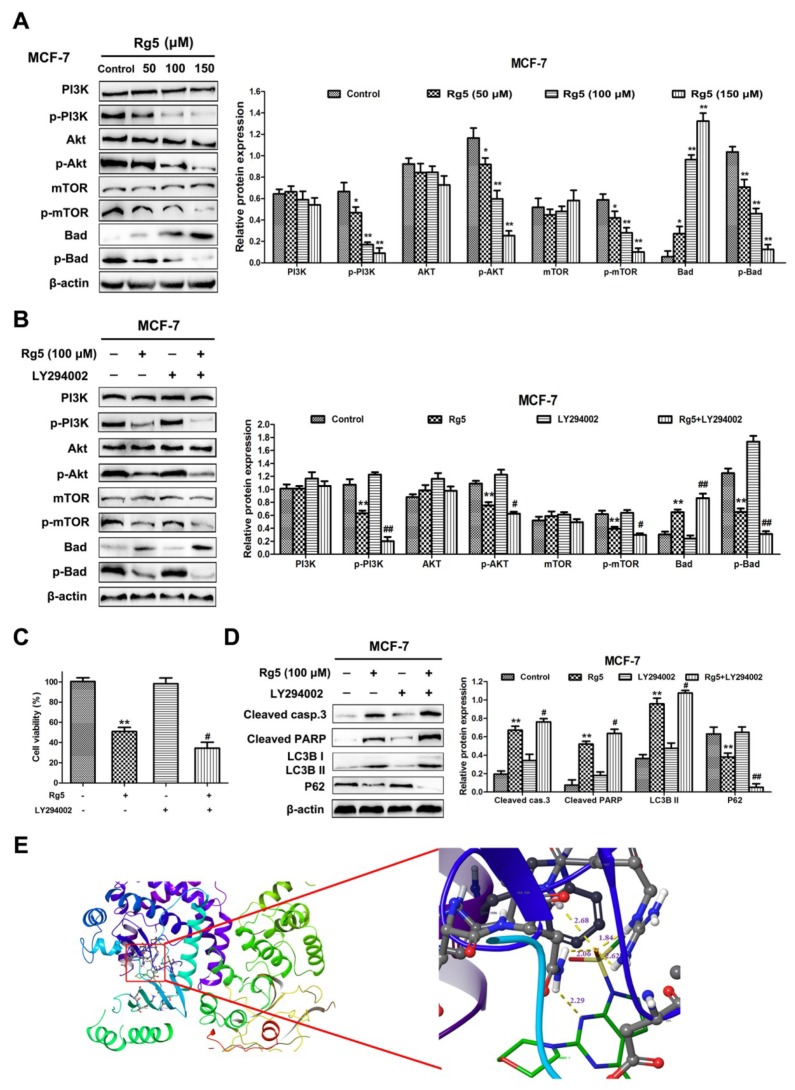
Rg5 induces apoptosis and autophagy through inhibiting PI3K/Akt signaling pathways. (**A**) MCF-7 cells were treated with Rg5 at various concentrations for 24 h. The levels of PI3K, p-PI3K, Akt, p-Akt, mTOR, p-mTOR, Bad, and p-Bad were analyzed using Western blot analysis. (**B−D**) MCF-7 cells were exposed to Rg5 (100 μM) in the presence of LY294002 (20 μM) (**B**) The protein expression levels of members of the PI3K/Akt pathways were determined through Western blot analysis. (**C**) Cell viability was detected through MTT assay. (**D**) Western blot assay was performed to analyze the levels of cleaved caspase-3, cleaved PARP, LC3B II, and p62. (**E**) Predicted binding model of Rg5 with PI3K (docking score (S) is -10.8). Hydrogen bonds are shown as yellow dashed lines. Images are visualized by PyMoL. * *p* < 0.05 and ** *p* < 0.01 as compared with the control; ^#^ *p* < 0.05 and ^##^ *p* < 0.01 as compared with Rg5-treated cells.

To explain the mechanism by which Rg5 suppresses PI3K signaling pathways in breast cancer cells, the protein levels of PI3K, Akt, mTOR, and Bad and their phosphorylation were explored via Western blot assay. As observed in Figure 8A, the Rg5 treatment resulted in the downregulation of phosphorylated PI3K (Tyr607), Akt (Ser473), mTOR (Ser2448), and Bad (Ser136) in a concentration-dependent fashion. Nevertheless, there was no obvious change in the total expression levels of PI3K, Akt, and mTOR in MCF-7 cells (Figure 8A). Meanwhile, the total expression level of Bad was concentration-dependently increased in cells after Rg5 treatment (Figure 8A).

Subsequently, to further verify whether Rg5-induced apoptosis and autophagy are interrelated with the PI3K signaling pathways in MCF-7 cells, we pretreated the cells with LY294002 (a PI3K inhibitor) prior to Rg5 treatment. Figure 8B indicates that LY294002 markedly reduced the phosphorylation levels of PI3K, Akt, mTOR, and Bad and enhanced the level of Bad induced by Rg5. Additionally, Figure 8C revealed that pretreatment with LY294002 further enhanced cell death induced by Rg5. The Western blot results show that LY294002 significantly strengthened the expression of cleaved caspase-3, cleaved PARP, and LC3B-II and weakened the expression of p62 after Rg5 exposure (Figure 8D). Collectively, these findings suggest that Rg5 induces apoptosis and autophagy by suppressing the PI3K signaling pathway in breast cancer cells.

### 3.8. Molecular Docking Study and ADMET Prediction

To predict the possible binding mode of Rg5 with PI3K, we performed a molecular docking study. The crystal structure of PI3K (PDB code: 3APC) was downloaded from the Protein Data Bank for the docking calculations. As shown in Figure 8E, Rg5 interacts with residues His945, Arg947, and Asn951 through hydrogen bond interactions in the active pocket of PI3K (docking score S is −10.8). Taken together, the above results indicate that Rg5 clearly occupies the binding site of PI3K and displays vital molecular docking interactions with contiguous amino acids.

Furthermore, the predicted ADMET (absorption, distribution, metabolism, excretion, and toxicity) score is tabulated in Table S1. Rg5 showed a negative result for blood brain barrier (BBB) criteria, predicting that it cannot pass through the BBB. The pharmacodynamic behavior of drugs depends on its uptake and distribution and can possibly be ascertained by plasma protein binding (PPB). Here, the molecules showed medium binding affinity. Additionally, there was nonchronic toxicity, a noninhibitory feature for human ether-a-go-go-related gene (hERG), nondevelopmental toxicity, nonreproductive toxicity, nonhepatotoxicity, and no AMES toxicity, which suggests that Rg5 is relatively harmless for oral administration.

## 4. Discussion

Major and abundant ginsenosides are present in ginseng (e.g., Rb1, Rd, Re, and Rg1) with low biological activities [22]. Hence, it is critical to transform major ginsenosides into rare ginsenosides, which possess marked pharmaceutical activity and can be conveniently absorbed by the human body [18]. In our study, we first converted the major ginsenoside Rb1 to Rg3 by enzymatic bioconversion and, then, transformed ginsenoside Rg3 to Rg5 with acid-assisted high-temperature and high-pressure processing, as Rg5 is a rare ginsenoside that is more active in ginseng. Furthermore, previous studies have highlighted that Rg5 performs an effective and desirable approach for the treatment of cancers [17]. In this study, Rg5 exhibited an evident antitumor effect through apoptosis and autophagy mediated by the PI3K/Akt signaling pathway in human breast cancer.

Type I cell death, also called apoptosis, and type II cell death, also called autophagy, represent two morphologically different patterns of programmed cell death [23]. Apoptosis is characterized by cell membrane blebbing, i.e., the loss of mitochondrial membrane potential and chromosomal DNA fragmentation [24,25]. Recently, many investigators have revealed that the regulation of apoptotic cell death is involved in human breast cancer [26,27]. Our studies verified that Rg5 increased the proportion of apoptotic cells, as evidenced by AO/EB and Annexin V-FITC/PI staining assays. Moreover, according to the results of the qRT-PCR and Western blot assays, Rg5 could regulate apoptosis-related mRNA and proteins in a concentration-dependent way. Moreover, multiple mitochondrial stimuli play a vital role in the cell apoptotic signaling cascade [28,29]. In our research, JC-10 and MitoTracker Green staining assays demonstrated that Rg5 could reduce the MMP and damage the mitochondria in breast cancer cells. Furthermore, previous reports have shown that caspases are also crucial components of the apoptotic pathway [30]. Our Western blot results confirmed that Rg5 treatment clearly promoted the cleavage of caspase-3, caspase-8, caspase-9, and PARP. Taken together, Rg5 induced caspase-dependent apoptosis through the mitochondria-mediated pathway in breast cancer cells.

The other type of programmed cell death, autophagy, is a dynamic course involving the encapsulation of cytoplasmic portions and intracellular organelles in vacuoles called autophagosomes, which merge with lysosomes to form autophagolysosomes [31]. It has been reported that autophagy-related proteins containing LC3-II, P62, Beclin-1, Atg5, and Atg12 are also tightly correlated with autophagosome maturation and are generally used as crucial markers for evaluating autophagy [32,33,34]. In this work, Rg5 induced autophagy in breast cancer cells, which was demonstrated by the formation of autophagosomes observed by TEM, the upregulation of LC3-II, Beclin-1, Atg5, and Atg12 expression and the downregulation of p62 expression. Characterized by two main features, namely, the involvement of cytoplasmic material and culmination with lysosomal degradation, autophagy is a multistep process [35]. Rg5 treatment increased the level of LC3B puncta, and microscopic evaluation of tandem GFP-RFP-tagged LC3B in MCF-7 cells demonstrated that Rg5 induced the occurrence of autophagic flux. Furthermore, autophagy can be limited due to the delayed trafficking of autophagosomes to lysosomes and the fusion of autophagosomes and lysosomes [36,37]. Immunofluorescence analyses from LysoTracker Red staining with GFP-LC3B were used to establish that Rg5 treatment increased the formation of autophagolysosomes and led to the fusion of autophagosomes and lysosomes.

Moreover, in several cellular conditions, autophagy can promote cell death; in contrast, under other conditions, autophagy can protect cell survival [38,39]. Our research confirmed that the autophagy inhibitor 3-MA attenuated the inhibitory effect of Rg5 on MCF-7 cells, which demonstrated that autophagy induced by Rg5 plays a role in contributing to cell death in breast cancer cells.

It is well known that the PI3K/Akt pathway is crucial for cell signaling and regulates cell survival, proliferation, and differentiation, especially in the progression of breast cancer [40,41]. A growing amount of evidence has clarified that mTOR is the center of autophagic regulatory cascades, which directly associate with the PI3K/Akt pathway in the autophagic pathway [42,43]. Moreover, previous studies have confirmed that the PI3K/Akt signaling pathway can inhibit apoptosis in breast cancer by regulating the phosphorylation of Bad [44,45]. Cell death can be promoted through Bad binding with Bcl-2 but blocked by Bad phosphorylation [44,45]. In the current study, Rg5 reduced the phosphorylation of PI3K, Akt, mTOR, and Bad, which suggested that Rg5 suppressed the PI3K/Akt pathway in breast cancer cells. Additionally, Western blot results implied that pretreatment with LY294002 dramatically regulated the expression of apoptosis- and autophagy-related proteins induced by Rg5. Furthermore, molecular docking demonstrated that Rg5 could form hydrogen bonds with His945, Arg947, and Asn951 in the active pocket of PI3K. Collectively, these results demonstrated that Rg5 could trigger apoptosis and autophagy by regulating the PI3K/Akt pathway while tightly binding to PI3K in breast cancer cells.

## 5. Conclusions

**Figure 9:**
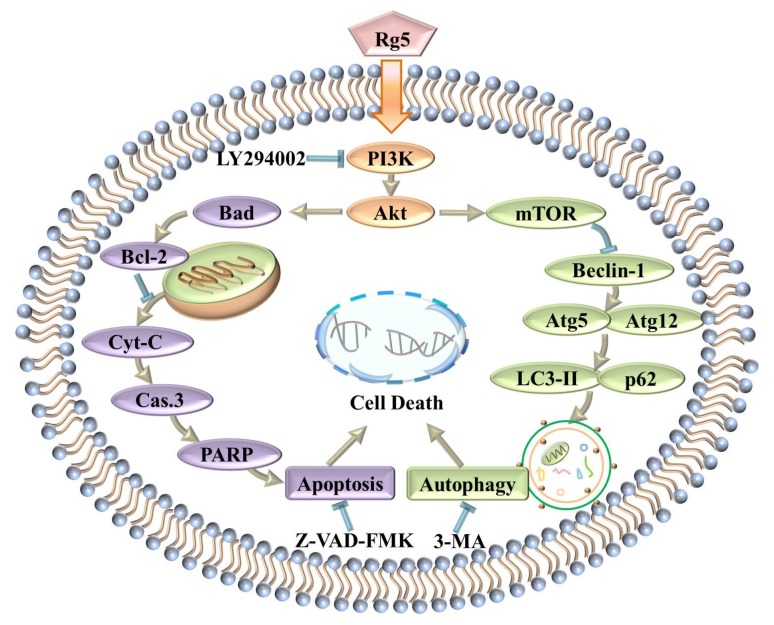
Schematic representation of the hypothesized molecular mechanism underlying the anti-breast cancer activity of Rg5.

In summary, this study implied that ginsenoside Rg5 exhibited the most potent antiproliferative activity against breast cancer among the various cancer cell lines tested. Furthermore, Rg5 inhibited cell proliferation by inducing apoptosis and autophagy in MCF-7 cells. Moreover, the antitumor mechanism of Rg5 was associated with suppressing the PI3K/Akt signaling pathway to trigger apoptosis and autophagy (Figure 9). Finally, molecular docking analysis confirmed that Rg5 regulated the function of breast cancer cells targeting PI3K. These findings demonstrated that Rg5 could be a novel and valuable antitumor agent targeting breast cancer.

## Figures and Tables

**Table 1:** Information on the antibodies used for Western blot analysis.

Antibody	Working Dilution	Molecular Weight (KDa)	Cat. Number
Cleaved caspase-3	1:1000	17	AB29034
Cleaved caspase-8	1:1000	47	AB40502
Cleaved caspase-9	1:1000	17	AB40503
Cleaved PARP	1:1000	85	AB29033
Bax	1:5000	21	50599-2-Ig
Bcl-2	1:2000	26	12789-1-AP
Atg5	1:1000	55	10181-2-AP
Atg12	1:1000	48	11122-1-AP
Beclin-1	1:2000	60	11306-1-AP
p62	1:2000	62	66184-1-Ig
LC3B	1:4000	16, 18	AB51520
Cytochrome c	1:1000	14	4280
β-actin	1:4000	42	20536-1-AP
PI3K	1:1000	110	20584-1-AP
phospho-PI3K	1:1000	85	ABP50495
AKT	1:1000	60	AB21054
phospho-AKT	1:1000	60	AB11054
mTOR	1:1000	289	AB21214
phospho-mTOR	1:1000	289	AB11221
Bad	1:2000	18	10435-1-AP
phospho-Bad	1:1000	23	AB11068
Goat anti-Rabbit IgG	1:20000	—	A21020

**Table 2:** Sequences of the primers used for quantitative real-time polymerase chain reaction (qRT-PCR).

Genes	Forward Primer (5′–3′)	Reverse Primer (5′–3′)
β-Actin	ttccagccttgcttcctg	tacttgcgcttgggagga
Bax	cccccgagaggtcttttt	tcccggaggaagtccaat
Bcl-2	aatgtgcccagcctcttg	tctgttgcccaactgcaa
LC3B	atgttgccacctcccaaa	tccagcacgagttcacga
P62	agcgggcatcagtttgag	gccctcctttccgatgat

## Supplementary Materials

The following are available online at https://www.mdpi.com/2072-6643/12/1/246/s1, Table S1: ADMET property evaluation, Figure S1: Thin layer chromatography (TLC) analysis of time-course transformation of ginsenoside Rb1 by β-glucosidase. S is the mixture of ginsenoside standards, Figure S2: Thin layer chromatography (TLC) analysis of time-course transformation of ginsenoside Rg3 at 121 °C with high pressure processing. S is the mixture of ginsenoside standards.
