# Supplementary material for: The Preparation of Ginsenoside Rg5, Its Antitumor Activity against Breast Cancer Cells and Its Targeting of PI3K
Source: Nutrients. 2020 Jan 18;12(1):246. doi: 10.3390/nu12010246 (PMC7019936; doi:10.3390/nu12010246)
Supplement: Supplementary file 1 [file nutrients-12-00246-s001.pdf]

Supplementary Information for

The preparation of ginsenoside Rg5, its antitumor activity against breast cancer cells and its targeting of PI3K

Yannan Liu[a,b,c], Daidi Fan[a,b,c*]

[a]Shaanxi Key Laboratory of Degradable Biomedical Materials, School of Chemical Engineering, Northwest University, 229 North Taibai Road, Xi'an, Shaanxi 710069,China

[b]Shaanxi R&D Center of Biomaterials and Fermentation Engineering, School of Chemical Engineering, Northwest University, 229 North Taibai Road, Xi'an, Shaanxi 710069, China

[c]Biotech. & Biomed. Reserch Institute, Northwest University, Taibai North Road 229, Xi'an 710069, Shaanxi, China.

Correspondence: Daidi Fan *E-mail addresses: fandaidi@nwu.edu.cn

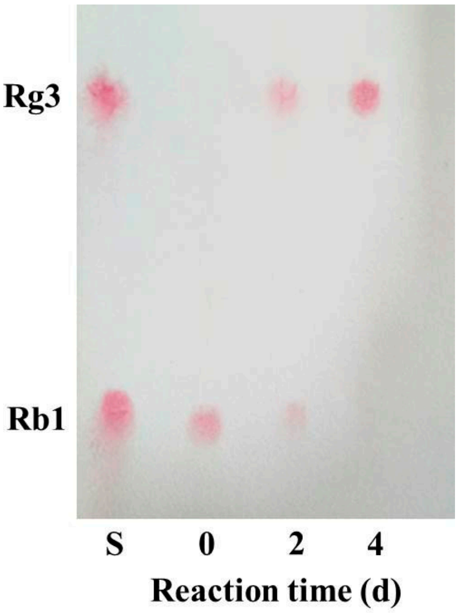

Supplementary Figure S1. Thin layer chromatography (TLC) analysis of time-course transformation of ginsenoside Rb1 by β-glucosidase. S is the mixture of ginsenoside standards.

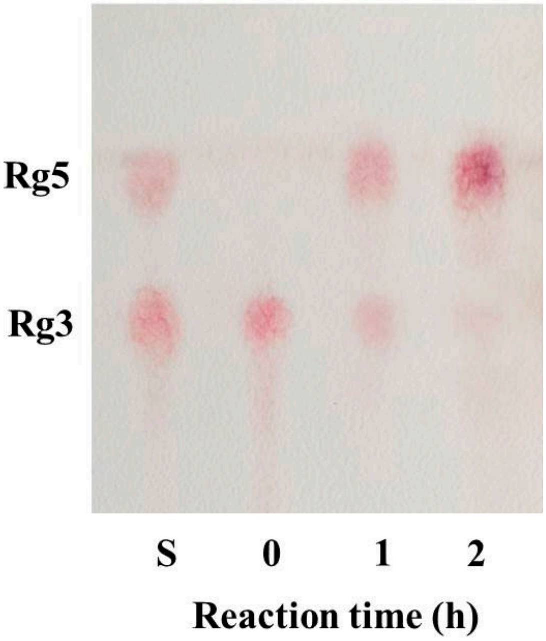

Supplementary Figure S2. Thin layer chromatography (TLC) analysis of time-course transformation of ginsenoside Rg3 at 121°C with high pressure processing. S is the mixture of ginsenoside standards.

Supplementary Table S1. ADMET property evaluation

Drug	BBB (%)	PPB (%)	Chronic toxicity(%)	hERG inhibition	Developmental toxicity	Reproductive toxicity	Hepatotoxicity	AMES toxicity
Rg5	Non (82.47)	81.41	Non (73.67)	Non (75.01)	Non (98.28)	Non (91.86)	Non (71.45)	Non (91.65)
